# A Prerecognition Model for Hot Topic Discovery Based on Microblogging Data

**DOI:** 10.1155/2014/360934

**Published:** 2014-08-26

**Authors:** Tongyu Zhu, Jianjun Yu

**Affiliations:** ^1^State Key Laboratory of Software Development Environment, Beihang University, Beijing 100191, China; ^2^Computer Network Information Center, Chinese Academy of Sciences, Beijing 100190, China

## Abstract

The microblogging is prevailing since its easy and anonymous information sharing at Internet, which also brings the issue of dispersing negative topics, or even rumors. Many researchers have focused on how to find and trace emerging topics for analysis. When adopting topic detection and tracking techniques to find hot topics with streamed microblogging data, it will meet obstacles like streamed microblogging data clustering, topic hotness definition, and emerging hot topic discovery. This paper schemes a novel prerecognition model for hot topic discovery. In this model, the concepts of the topic life cycle, the hot velocity, and the hot acceleration are promoted to calculate the change of topic hotness, which aims to discover those emerging hot topics before they boost and break out. Our experiments show that this new model would help to discover potential hot topics efficiently and achieve considerable performance.

## 1. Introduction

Microblogging (post) is a mini blog which is typically smaller in both actual and aggregate file size comparing with a traditional blog. Microblogging allows users to exchange small elements of content such as short sentences, individual images, or video links. As a convenient communication means, especially with mobile phone, microblogging has been prevailing in the Internet. Sina Weibo (a Chinese Twitter) produces 25,000,000 messages each day, and Twitter gets 50,000,000 for each day.

In our opinion, there are two main reasons that bring the bloom of microblogging. The first reason is the initiative of posting concerning messages of each person ranging from the simple such as “what I'm doing right now” to the thematic such as political theme. The second reason is that the mobile phone would help users to utilize the splitting time to concern the topics on the microblogging systems.

With a large amount of reading and communication from users, it is quite understanding that hot topics would show up since most of people are concerned about those emergent incidents, such as “missing flight MH370.” Of course there are a lot of rumors since Internet is anonymous. It is a good way for local government and department to publish latest news about their work to dismiss rumors. However we argue that it is more important to discover those hot topics in advance. That means we need to construct a prerecognition model for hot topic discovery.

Most of current work usually focuses on the postrecognition of hot topic discovery for analysis with history dataset. They are difficult to check the real-time status of topics, which is unfavorable to control those rumors. In this paper, we emphasize our work on the prerecognition mechanism and propose a novel hot topic discovery system which integrates previous hot topic discovery mechanisms with the concept of hot velocity and hot acceleration to recognize potential hot topics before they boost and break out.

This paper aims to enhance our previous work on prerecognition of hot topic discovery [1]. We firstly promote a topic life cycle model that defines the different status of a topic from its appearance to its disappearance. Then we utilize the topic hot velocity and the hot acceleration borrowing from “mechanics field” to calculate the change of topic hotness, which aims to discover those hottest topics before they are hot ones. The prerecognition model helps to find those potential hot topics and checks the real-time status of each topic, which can be applied for local government to guide public opinion and build a harmonious society. Also it would help e-business enterprise to deliver customized advertisement for interested users.

The rest of the paper is organized as follows. In Section 2, we discuss related work. We give the related definitions at Section 3.  Section 4 provides our prerecognition model for hot topics.  Section 5 shows our experiment results. We present further discussion at Section 6. Finally, we conclude and discuss some future work.

## 2. Related Work

Hot topic prerecognition is basically to aggregate those similar microbloggings, formalize topic clusters, and then rank topic clusters with the count of included posts, the hot velocity, and the hot acceleration.

### 2.1. Topic Detection

Much work has been done for topic discovery before microblogging's appearance. TDT (Topic Detection and Tracking) is one of the popular approaches. TDT aims to discover the topical structure in unsegmented streams of news reporting as it appears across multiple media and in different languages. Since hot topic discovery is focusing on real-time topic stream nowadays, we would like to introduce those online models. TID (Topic Initiator Detection) [2] introduced a web mining and search technique for a specificized topic query and gave resulting collection of time-stamped web documents which contain the query keywords. Petrovic et al. provided a similar work [3] to detect new events from a stream of Twitter posts. In particular, they gave comparison with other systems on the first story detection task. Pan and Mitra introduced two event detection approaches using generative models [4]. They combined the popular LDA (Latent Dirichlet Allocation) model with temporal segmentation and spatial clustering and adapted an image segmentation model, SLDA (Supervised Latent Dirichlet Allocation), for spatial-temporal event detection on text. Since finding and clustering topics with generative models like LDA and its extension, we would adopt LDA series as our topic model for clustering. Other work on online news detection and tracking was introduced in papers [5–7]. In our opinion, these papers focused more on topic discovery for traditional messages, such as posts from forums and blogs. The original dataset of microblogging is larger than those traditional datasets, and it is real-time stream. Therefore, how to detect topics on large scale of stream texts has been hot research topic in recent years.

### 2.2. Topic Discovery with Combined Features

Current work on emerging topic discovery with microblogging always applied several features of posts, such as textual information, graph connection, and the time factor to find those emerging topics.

As for using textual information feature, Kasiviswanathan et al. identified emerging topics through detection and clustering of novel user-generated content in the form of blogs, microbloggings, forums, and multimedia sharing sites with dictionary learning approach [8]. Goorha and Ungar described a system that monitored social and mainstream media to determine shifts in what people are thinking about, a product or company [9]. Bai et al. provided hot events detection based on burst terms, terms co-occurrence, and generative probabilistic model [10]. Jo et al. defined a topic as a quantized unit of evolutionary change in content and discovered topics with the time of their appearance in the corpus to capture the rich topology of topic evolution inherent [11]. These work focused on the text clustering and the topic model utilization. They considered little on the feature of the topic increasing rate.

Considering the time factor, Zhu et al. proposed a method for discovering the dependency relationship between the topics of documents in adjacent time stamps based on the knowledge of content semantic similarity and social interactions of authors and repliers [12]. Iwata et al. proposed an online topic model for sequentially analyzing the time evolution of topics in document collections considering both the long-timescale dependency and the short-timescale dependency [13]. Yin et al. detected both stable and temporal topics simultaneously and provided a unified user-temporal mixture model to distinguish temporal topics from stable topics [14].

Besides the time factor, some researchers thought that the graph connection could be one of the important sources to detect emerging topics. Cataldi et al. made use of a term aging model to compute the burstiness of each term and provided a graph-based method to retrieve the minimal set of terms that can represent the corresponding topic [15]. Zhou and Chen proposed a graphical model called location-time constrained topic (LTT) to capture the content, time, and location of social messages for event detection [16]. Zhao et al. used a subspace clustering algorithm to group all the social objects into topics and then divided the members that are involved in those social objects into topical clusters, each corresponding to a distinct topic [17].

Some other work combined more features for topic detection. Chen et al. [18] crawled the relevant messages related to the designated organization by monitoring multiple aspects of microblog content, including users, the evolving keywords, and their temporal sequence. They then developed an incremental clustering framework to detect new topics and employed a range of content and temporal features to help in promptly detecting hot emerging topics. Moreover, emerging topic detection technologies are widely applied for diverse applications, such as earthquake reporting [19], location-specific tweet detection [20], and geospatial event detection [21].

### 2.3. Summary

Tu and Seng [22], He and Parker [23] proposed similar ideas of our model. Tu and Seng provided a new set of indices for emerging topic detection. They defined novelty index (NI) and the published volume index (PVI) to determine the detection point (DP) of new emerging topics, which used ACM Digital Library as experimental data. He and Parker reconstructed bursts as a dynamic phenomenon using kinetics concepts from physics (mass and velocity) and derived momentum, acceleration, and force from the concepts. Also they referred to the result as topic dynamics, permitting a hierarchical, expressive model of bursts as intervals of increasing momentum. They used PubMed/MEDLINE database of biomedical publications as experimental data.

Different from these models, we define the topic life cycle, the hot velocity, and the hot acceleration to recognize hot topics and use the microblogging dataset to examine our model. And our goal is to find those hot topics in advance. So in this paper, we combine the concept of topic model with the topic life cycle to define a prerecognition model for emerging topic detection.

## 3. Definition

Before introducing the prerecognition model, we would like to give some related definitions for hot topic discovery.

**Definition 1 (post).**

A post (microblogging) *p* is an original message crawled from a microblogging system published by a user *u*, which can be expressed as *p* = {*p*∣*w*_1_, *w*_2_,…, *w*_*n*_, *n* ≤ 20}.

The original message of a post always includes text, video link, audio link, images, retweet, and comment information. In this paper, we focus more on textual content in a post which inspires us to define the post *p* as a sequence of keywords *w* from the view of NLP (Natural Language Processing). Since a post is always limited with the word count (most of microblogging systems maximize the word count to 140), we assume that the maximum count of keywords *w* of a post is 20. Considering the particularity of the Chinese microblogging system, we generated the Chinese keywords from several basic corpus, including Sogou Pinyin input dict (http://pinyin.sogou.com/dict/), NLPIR microblogging corpus (http://www.nlpir.org/).

**Definition 2 (topic).**

A topic to is what posts are talking about and is composed of a set of posts. A topic may include a set of subtopics; thus it can be expressed as to = {to∣to_1_, to_2_,…, to_*k*_, *p*_1_, *p*_2_,…, *p*_*j*_, *k* ≥ 0, *j* > 0}.

Always a new topic is generated from a series of posts, whereas, with its evolution, a topic may derive subtopics which are discussing about the same theme but with partly distinct keywords. Of course a subtopic to_*i*_ may derive sub-subtopics to_*im*_ until a subtopic becomes a new topic representing totally different theme and cannot be derived at that time.

**Figure 1:**
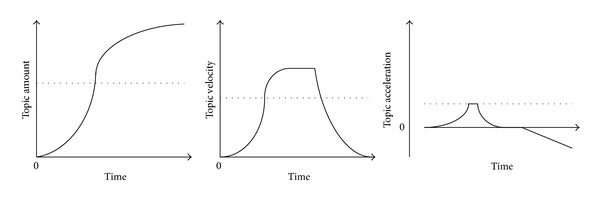
A hot topic with the topic amount, velocity, and acceleration.

As we observed, when a topic is becoming a hot topic, the following conditions should be satisfied: (1) the topic amount is high enough, which means the number of posts included in the topic exceeds a predefined threshold; (2) the speed of the topic amount is high enough, which shows that the topic amount should increase quickly in a short time; (3) the acceleration of topic increment grows fast.  Figure 1 gives an example of a hot topic with its amount, velocity, and acceleration. Thus we define three concepts to identify a hot topic: the topic amount, the topic hot velocity, and the topic hot acceleration.

**Definition 3 (topic amount).**

Topic amount ∑_*t*_to describes how many posts *p* belong to current topic and its subtopics: ∑to = ∑^to_*i*_^∑*p*_*ij*_ + ∑*p*_*j*_.

**Definition 4 (topic hot velocity).**

Topic hot velocity thv is to express how fast a topic to increases, which is calculated with topic amount in a period time *t*: thv = ∑_*t*_to/*t*, *t* > 0, *t*%Δ*t* = 0.

Δ*t* is the minimized time period to process the original posts and get the topics.

**Definition 5 (topic hot acceleration).**

Topic hot acceleration tha shows the speed of thv, which can be presented as the first derivative of topic hot velocity tha = thv′.

As we have observed, when a topic is emerging, tha always gets high, which would be an important metric to determine whether a topic is hot or not.

As shown in Figure 1, we can find that a topic exists significant patterns from appearance to disappearance, which inspire us to put forward the concept of the topic life cycle.

**Definition 6 (topic life cycle).**

A topic life cycle defines topic status, which offers six status tlc = {embryo, boost, outbreak, stabilization, recession, extinction}.

**Figure 2:**
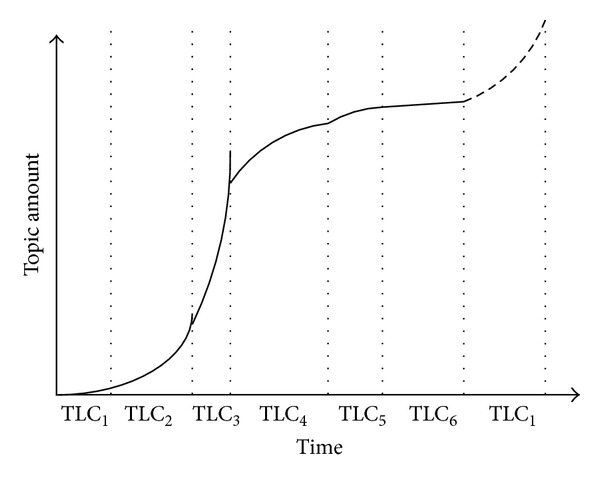
A topic life cycle with different periods.

In our opinion, a topic life cycle includes six periods: embryo, boost, outbreak, stabilization, recession, and extinction as shown in Figure 2. A topic shows up when people begin to discuss about it, in which stage we call embryo presented as TLC_1_. In this period, the topic amount is increasing slowly. When more people begin to concentrate on a specialized topic, the topic amount would increase in a very short time, in which stage we call boost presented as TLC_2_. In this period, the thv and tha are increasing continuously which makes this topic be a potential hot topic. When the topic amount and the thv are increasing continuously whereas the tha is increasing not so fast, in which stage we call outbreak presented as TLC_3_. In this period, the thv would achieve its maximum value. When the thv has a relatively fixed value, we call this period stabilization presented as TLC_4_. When a topic decreases quickly in a short time, we call this stage recession presented as TLC_5_. When a topic is almost not discussed, we call this period extinction presented as TLC_6_.

Also a topic life cycle has its periodicity according to the evolution of attention from the public, which means a topic may have several life cycles consequently.

**Definition 7 (transformation point).**

A transformation point is the point between different periods of a topic life cycle expressed as tp = {tp∣tlc_*i*_∩tlc_*j*_}.

The transformation point shows the status change for different periods of a topic life cycle. Thus we get five transformation points for a complete topic life cycle consequently.

According to the above definitions, we can describe a hot topic as follows.

**Definition 8 (hot topic).**

A hot topic would always be in the period of boost and the topic amount should exceed the threshold *ξ* expressed as hotto = {hotto∣to ∈ boost, tp ∈ {boost∩outbreak}, ∑to ≥ *ξ*}.

With the above definitions, we then offer the prerecognition model in detail at the next section.

## 4. Prerecognition Model

As described above, different from other works on hot topic discovery, our contributions on hot topic discovery can be summarized as follows.

1. The first one is that our model aims to find those emerging topics before they are hot ones since we apply a prerecognition model, which can catch the instant changes of the topics on their topic amounts, topic velocity, and topic acceleration.
2. We borrow the concepts of “velocity” and “acceleration” from physics, which can well illustrate the dynamics of the hot topics.
3. We define the concept of topic life cycle, which can capture the periodic characteristics of hot topics. Moreover, calculating the ∑to ≥ *ξ* during the period of boost brings the success of the prerecognition model.

### 4.1. Prerecognition Steps

The prerecognition model is to find those potential hot topics with ∑to ≥ *ξ* during the period of boost in a topic life cycle; thus three processes should be followed.

1. Clustering the original posts to get topics and their amount: we also extend this process into five steps: filtering the original posts to omit the stop and useless words, matching the preprocessed words to get keywords, using LDA [24] and PAM (Pachinko Allocation Model) [25] topic model to generate topic and its subtopics, and finally clustering similar topics and getting their amounts using KNN (K-Nearest Neighbor) algorithm.
2. Calculating the velocity and acceleration of the topic: we define several transformation points, threshold of thv and tha, to find the different periods of the topic life cycle.
3. Selecting potential hot topics during the boost period through checking their ∑to, thv, and tha.

### 4.2. Topic Clustering

The topic clustering step aims to classify streamed posts into different topics. We should first collect original posts from different microblogging systems, for example, Sina (http://weibo.com/), QQ (http://t.qq.com/), and Twitter (http://twitter.com/). We develop a crawler gathering posts' textual information with open APIs provided by these microblogging systems. It is important to note that a post may include hashtag which is a manually labeled hashtag expressed with #xx# (xx represents word term). In this paper, we extract #xx# as a topic directly since this token can express the semantics explicitly. For the other plain text, we need to extract keywords from the posts and then cluster the current keywords to generate topics. As we observed, a post can be viewed as a series of keywords that delivers the similar scenario of topic model. Topic model schemes each post as a mixture of topics, and each topic is a multinomial distribution over words in a vocabulary, which inspires us to introduce the topic model for post clustering. LDA is one of the increasingly popular tools for summarization and discovery with the capability of automatically extracting the topical structure of large document collections. LDA constructs a three-level hierarchical Bayesian model based on the idea of topics. Each document exhibits multiple topics with different proportions, and the topic proportions are document-specific and randomly drawn from a Dirichlet distribution. Each topic is also modeled as an infinite mixture over a set of words probabilities.

We use LDA to sample each post with multinomial dirichlet distribution over topics, and then repeatedly sample each topic with multinomial distribution over keywords as expressed in

(1) pD ∣ α,β  =∏d=1M∫pθd ∣ α    ·∏n=1Nd ∑zdnpzdn ∣ θdpwdn ∣ zdn,βdθd.

In the three-level Bayesian network of LDA, parameters *α* and *β* are applied to corpus level where *α* is a *k* vector and *β* is a *k* × *V* matrix (*k* is the the dimensionality of the topic variable *z*, and *V* is the length of a keywords vector from a vocabulary). *θ*_*d*_ is a document level variable which presents multinomial distribution over topic *z*_*n*_ and ∑_*i*=1_^*k*^ *θ*_1_ = 1. The *z*_*dn*_ and *w*_*dn*_ are word level variables which measure the multinomial probability of a word *w*_*n*_ in document *d* with a topic *z*_*n*_.

LDA topic model helps to capture the correlations among words and improve the recall of topic discovery. However, it does not explicitly model correlations among topics; that is, topics are not just plain textual documents but present strong structural information among topics. The ignored correlations among topics limit LDA's ability to mine the underling context of topic [26]. In this paper, we will model the hierarchically structural information to reveal the correlations among topics by PAM approach. PAM [26] uses a directed acyclic graph (DAG) structure to represent and learn arbitrary-arity, nested, and possibly sparse topic correlations. In PAM, the concept of topics is extended to be distributions not only over words, but also over other topics, that is, subtopics.

To got a post, PAM samples *θ*_*t*_1__^(*d*)^, *θ*_*t*_2__^(*d*)^,…, *θ*_*t*_*s*__^(*d*)^ from *g*_1_(*α*_1_), *g*_2_(*α*_2_),…, *g*_*s*_(*α*_*s*_). *θ*_*t*_*i*__^(*d*)^ is a multinomial distribution of topic *t*_*i*_ over its children. *r* is the parent of all topic nodes and is associated with a Dirichlet distribution *g*(*α*). For each word *w* in the post, PAM samples a topic path *z*_*w*_ of length *L*_*w*_ : 〈*z*_*w*1_, *z*_*w*2_,…, *z*_*wL*_*w*__〉. *z*_*w*1_ is the root, and *z*_*w*2_,…, *z*_*wL*_*w*__ are topic nodes in *T*. *z*_*wi*_ is a child of *z*_*w*_(*i* − 1) and it is sampled according to the multinomial distribution *θ*_*z*_*w*(*i*−1)__^(*d*)^. And then sample word *w* from *θ*_*z*_*wL*_*w*___^(*d*)^. Then PAM gets

(2) Pd,zd,θd ∣ α=∏i=1sPθtid ∣ αi·∏w∏i=2LwPzwi ∣ θzwi−1dPw ∣ θzwLwd.

With *θ* ^*d*^ and *z* ^(*d*)^, PAM calculates the marginal probability of *d* as

(3) Pd ∣ α  =∫∏i=1sPθtid ∣ αi     ·∏w ∑zw∏i=2LwPzwi ∣ θzwi−1dPw ∣ θzwLwddθd.

Finally, the probability of generating whole posts is a product of the probability for each post:

(4) $$\begin{array}{c} P\left(D\;|\;\alpha \right)=\underset{d}{\prod }P\left(d\;|\;\alpha \right). \end{array}$$

With (1)–(4), we can calculate the relation between different topics using generative model, which helps us to classify similar topics and calculate the topic amount of included posts applying KNN (K-Nearest Neighbor) algorithm.

### 4.3. Calculating Topic Parameters

We need to calculate three parameters for a topic: topic amount, topic hot velocity, and hot acceleration. Considering the time duration of topic clustering, we set the time interval Δ*t* as 1 hour. That means we will cluster posts and calculate topic parameters at each hour.

According to the definition of hot velocity and hot acceleration, we get the instants $\tilde{\text{thv}}$ and $\tilde{\text{tha}}$, averages $\bar{\text{thv}}$ and $\bar{\text{tha}}$. Consider

(5) $$\begin{array}{c} \tilde{\text{th}{\text{v}}_{\text{t}{\text{o}}_{n}}}=\overset{t}{\underset{i}{\sum }}\left(\text{t}{\text{o}}_{n}\right)-\overset{t-\Delta t}{\underset{i}{\sum }}\left(\text{t}{\text{o}}_{n}\right). \end{array}$$

$\tilde{\text{th}{\text{v}}_{\text{t}{\text{o}}_{n}}}$ is measured with topic amount increment at time *t* and time *t* − Δ*t*, that is, the post increment of a topic after a time interval Δ*t*. Consider

(6) $$\begin{array}{c} \bar{\text{th}{\text{v}}_{\text{t}{\text{o}}_{n}}}=\frac{\left(\sum_{}^{k}\text{t}{\text{o}}_{n}-\sum_{}^{j}\text{t}{\text{o}}_{n}\right)\times \Delta t}{t_{k}-t_{j}}. \end{array}$$

$\bar{\text{th}{\text{v}}_{\text{t}{\text{o}}_{n}}}$ is measured with the topic amount increment at time *t*_*k*_ and time *t*_*j*_.

Similar to the calculating steps of the topic hot velocity, we get $\tilde{\text{th}{\text{a}}_{\text{t}{\text{o}}_{n}}}$ and $\bar{\left(\text{th}{\text{a}}_{\text{t}{\text{o}}_{n}}\right)}$ as follows:

(7) $$\begin{array}{c} \tilde{\text{th}{\text{a}}_{\text{t}{\text{o}}_{n}}}=\overset{t}{\underset{i}{\sum }}\left(\tilde{\text{th}{\text{v}}_{\text{t}{\text{o}}_{n}}}\right)-\overset{t-\Delta t}{\underset{i}{\sum }}\left(\tilde{\text{th}{\text{v}}_{\text{t}{\text{o}}_{n}}}\right). \end{array}$$

$\tilde{\text{th}{\text{a}}_{\text{t}{\text{o}}_{n}}}$ is measured with the thv increment after a time interval Δ*t*. Consider

(8) $$\begin{array}{c} \bar{\left(\text{th}{\text{a}}_{\text{t}{\text{o}}_{n}}\right)}=\frac{\left(\sum_{}^{k}\tilde{\text{th}{\text{v}}_{\text{t}{\text{o}}_{n}}}-\sum_{}^{j}\tilde{\text{th}{\text{v}}_{\text{t}{\text{o}}_{n}}}\right)\times \Delta t}{t_{k}-t_{j}}. \end{array}$$

$\bar{\left(\text{th}{\text{a}}_{\text{t}{\text{o}}_{n}}\right)}$ represents the thv increment in the interval from *t*_*j*_ to *t*_*k*_.

### 4.4. Hot Topic Recognition

**Figure 3:**
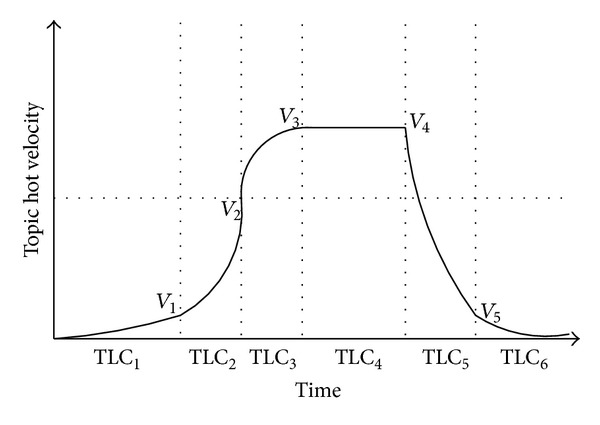
Topic life cycle illustrated with the topic hot velocity.

As described above, prerecognition model aims to find those topics before they become hot topics, so we should find which period a topic belongs to. In Figure 3, we give the characteristics of each period expressed with the topic hot velocity.

As shown in Figure 3, we can determine each period of topic life cycle through calculating the topic parameters.

If a topic is in its embryo stage, we can get the following equation:

(9) embryo=0<thv¯<V1,0<tha¯<α1,thv~<V1,tha~<α1.

The transformation point tp_1_ between embryo and boost can be calculated as follows:

(10) $$\begin{array}{c} \text{t}{\text{p}}_{1}=\left\{\tilde{\text{thv}}=V_{1},\tilde{\text{tha}}=\alpha_{1}\right\}. \end{array}$$

The boost period and tp_2_ can be calculated as follows:

(11) boost=V1<thv¯<V2,tha¯>α1,tp2=∑ton≥ξ,thv~=V2.

We record the $\tilde{\text{tha}}$ of tp_2_ as *α*_2_.

The outbreak period and tp_3_ can be calculated as follows:

(12) outbreak=V2<thv¯<V3,0<tha¯<α2,tp3=tha~=0.

The stabilization period and tp_4_ can be calculated as follows:

(13) stabilization=V4<thv¯<V3,tha¯≤0,tp4=thv~=V4.

We record the $\tilde{\text{tha}}$ of tp_4_ as *α*_3_.

The recession period and tp_5_ can be calculated as follows:

(14) recession=V5<thv¯<V4,tha¯≤α3,tp5=thv~=V5.

We record the $\tilde{\text{tha}}$ of tp_5_ as *α*_4_.

The extinction period can be calculated as follows:

(15) extinction=thv¯<V5,tha¯≤α4,thv~<V1,tha~<α1.

According to the calculating steps of each period of topic life cycle and corresponding transformation point, we can easily find those potential hot topics; that is, we can choose those potential hot topics at tp_2_. We then rank these potential hot topics as our results.

### 4.5. Recognition Algorithm

The following codes give the recognition algorithm for hot topic discovery: see Algorithm 1.

### 4.6. Complexity Analysis

For simplicity, we just omit the complexity of the clawers and only present the complexity of prerecognition model. The clustering steps include topic generation and cluster generation. As for topic generation, the total running time is *O*((*NT*)^*τ*^(*N* + *T*)^3^), where *N* is the number of words, *T* is the number of latent topics, and *τ* is the number of topics appearing in a post. According to our observation, the number of topics included in a post would be less than 3, which inspires us to set *τ* = 3 for few computational costs. As a result, the computational complexity of LDA is *O*(((*NT*)×(*N* + *T*))^3^). The complexity of PAM is similar to the LDA except its depth of children (topic level); that is, the computational complexity of LDA is *O*(*r* × ((*NT*)×(*N* + *T*))^3^), where *r* is the depth of children. In this paper, we set the maximum value of *r* = 8 for reducing the computational costs. As for cluster generation, the complexity is *O*(*T*), where *T* is the total number of topics.

The topic parameter calculation complexity is linearly related with the the number of posts; that is, the complexity is *O*(*n*), where *n* is the number of posts.

## 5. Experiments and Evaluation

We set a server cluster to evaluate the efficiency of our model. The cluster includes 10 PC servers, each server having 2 CPU, 32 GB memory, and 4 TB disk storage. We distribute 4 servers to collect the real dataset since the microblogging systems always limit the number of posts being crawled. The remaining servers are distributed for hot topic recognition. And all experiments are evaluated with 100 Mb bandwidth.

We crawled approximately 2,000,000 original posts from Sina, QQ microblogging systems with their APIs. The dataset contained 675,439 valid posts after preprocessing to filter those meaningless ones (those posts with less retweet count than 500) from 2014/01/01 to 2014/04/30. Of course this dataset cannot include all posts because of the limit of API. However, we investigated that it is enough to validate our model since the crawled posts would cover almost all concerned topics. We choose our training dataset from 2014/01/01 to 2014/01/31 and other posts as test dataset. In the training dataset, there are 208,563 posts and 903,772 words which are identified by 80,000 terms.

In our datasets, the topics and keywords are almost Chinese terms. Considering the particularity of Chinese microblogging system, we generate these Chinese terms from several basic corpora, including Sogou Pinyin input dict, NLPIR microblogging corpus. For those English keywords, we just use the standard corpus.

### 5.1. Topic Clustering

We first cluster topics from the training dataset. We use C implementation of variational EM for LDA provided by Princeton University (http://www.cs.princeton.edu/~blei/lda-c/). When training LDA parameters, we figured out 500 latent topics manually from the training posts and get the parameters *α* = 0.05 and *β* = 0.1.

Table 1 gives five sample topics and top ten keywords' distributions over them. Though most of posts are generated with Chinese keywords, we prefer to present English keywords just for convenience. We found that these ten words indicate the topics well, which shows what people are talking about and gets a latent topic from these words apparently.

We have observed that column 2 and column 3 present the similar topic which should be classified into one topic “missing Flight MH370” with PAM model. Also we investigated that column 1 and column 5 are talking about two different topics; however, they are related topics since the “Ukraine crisis” is one of the reasons of “Crimea independence.” In the second scenario, we would classify them as two topics for simplicity.

We then presented top 10 hot topics with their names (summarized manually), amount of posts, amount of subtopics, the maximum level of subtopics, and average recall/precision after clustering process.

As shown in Table 2, the ranked top ten topics discovered with our model are also hot topics discussed most by people at the Internet, which proved that our model can separate hot topics from all discussing topics correctly. We observed that a topic always embedded average 4–7 levels of subtopics. The subtopics at the same level with the same parent topic have similar keyword distribution since one topic is always an evolution version of another one. The difference is that these subtopics are more concerning about one profile of the parent topic.

Also we observed that the recall/precision of most topics is not very high, which means some posts are ambiguous to be classified into one topic. In this paper, we aim to discover those potential hot topics quickly; we would like to improve recall of the topic, which inspires us to classify a post into a topic when its possibility is over a threshold *τ* = 60%.

### 5.2. Hot Topic Recognition Time

We have summarized those hot topics with our clustering model; another problem is to find those potential hot topics in their transformation point tp_2_. We made the simulated evaluation with the testing dataset and got the predict time and the corresponding topic hotness shown in Table 3. Also we presented the predict time comparing with Google Trend (http://www.google.com/trends/) and Baidu Index (http://index.baidu.com/) (measured with query amount and normalized with time base Δ*t*).

We should emphasize that topic amount and thv are far less than the query amount of Google and Baidu. However, we emphasized our focus on the predicting time for emerging hot topics. We observed that our result of finding a hot topic is always quicker than the query from search engine; this is because posts and topics are always published on the microblogging systems nowadays, then noticed by Internet users and traditional medias, and finally searched by interested people with search engines.

In our experiment, we set the thresholds *ξ* = 5,000, *V*_1_ = 800, and *α*_1_ = 800 to rank the potential hot topics comparing the scale of our dataset. Also these thresholds would be applied for real-time hot topics prediction.

The rapidity of prerecognition model for hot topic would be very useful for acquiring online public opinion, which helps to control rumors and guide the public opinion. Moreover, the advertisers can use this model to promote customized advertises to different users.

## 6. Discussion

**Figure 4:**
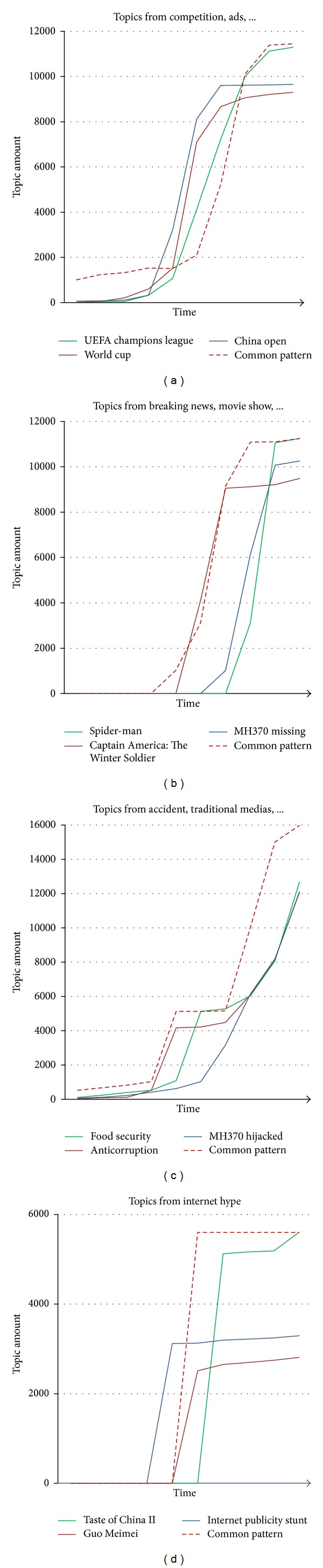
Topic hotness modes with different trends.

As we observed, different topics have their special trend models of becoming hot topics in their topic life cycles. As shown in Figure 4, we classified four types of topic hotness modes.

As shown in Figure 4(a), a hot topic increases slowly for a long time, then breaks out in a short time, and finally does not change the topic amount any more. In this mode, as we have observed, topics are from competition, ads, such as “China Open,” and football final match. These topics are always attractive for a long time before they show up and become the focus when they happen and disappear quickly when they finish. We can monitor those important events in advance since they are easy to recognize with almost fixed topic increasing model.

The second mode of topic hotness is shown in Figure 4(b). In this mode, a hot topic may be neglected by people for a long time, then break out in a short time, and finally disappear quickly and it does not change the topic amount any more. In this mode, topics are from breaking news, new movies, such as “MH370 missing” and “Captain America: The Winter Soldier”. These topics do not exist or are not discussed much before they show up and become the focus when they happen and disappear quickly when they finish.

The third mode of topic hotness is shown in Figure 4(c). In this mode, a hot topic increases slowly for a long time, then breaks out in a short time, and repeatedly will be few discussed in a long time, and finally breaks out again with more people involved in. In this mode, topics are from accidents with traditional medias involved in, such as “MH370 hijacked.” These topics do not exist or are not discussed much before they show up and become the focus when they happen. With more people participating in the topic and traditional medias pushing more concentration, the topic would be hotter and hotter.

The fourth mode of topic hotness is shown in Figure 4(d). In this mode, a hot topic breaks out suddenly and disappears quickly. In this mode, topics are from Internet news, such as “Internet publicity stunt.” These topics would exist for a very short time but present strong hotness.

According to the analysis of four modes of hot topics, we can capture important keywords to monitor hot topics in advance and control public opinion correspondingly.

**Figure 5:**
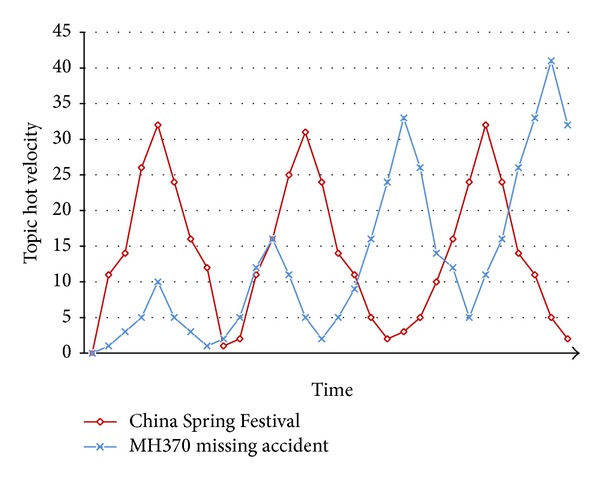
Topic trend with hot velocity in life cycles.

We have noticed that a hot topic may be hotter and hotter in the third mode. However, there exist two situations for periodic hot topics as shown in Figure 5.

A hot topic may present almost the same hot velocity at each life cycle for some special topics, such as “China Spring Festival.” These topics are concentrated at a fixed time and disappear when the events finish, and the concerned people and medias are always fixed. So the topic amount and topic hot velocity present the same speed at each life cycle correspondingly, which helps to capture these hot topics before the event shows up. Another scenario is that a hot topic may present higher hot velocity at each life cycle, such as “MH370 missing accident.” These topics would be focused on by more people with traditional medias involved in. When more and more people participate in the discussion, more and more posts are generated and certainly would result in higher topic velocity. In this scenario, any evidence or opinion may bring in more concentration.

The regular pattern of periodic hot topics would help us to monitor those periodic hot topics when they are in their periodic time or when new breaking news show up.

## 7. Conclusion

How to discover potential hot topics before they boost and break out on microblogging systems is a research focus, which also helps to guide rumor topics for government and deliver customized advertisement for e-business enterprise. In this paper, we scheme a novel prerecognition model for hot topic combining the generative model with the concept of the topic life cycle, the topic hot velocity, and the hot acceleration. We crawled test dataset from popular microblogging systems to verify our model. The experiments prompt that this prerecognition model can identify those emerging hot topics quickly.

Still there are several issues that should be solved. The first one is that a comparison between the model proposed in this paper and other similar models should be given to improve the persuasiveness. And then a parallel clustering algorithm should be provided to process large scale of posts. Another problem is how to portray the evolution of topics which may change the theme and keyword set of the topic to a large extent. Also in this paper, we omitted the factor of involved users, which may be an important metric to calculate the hotness of the topic. In our future work, we would analyze these issues further and do the corresponding experiments for our model.

## Figures and Tables

**Algorithm 1:**
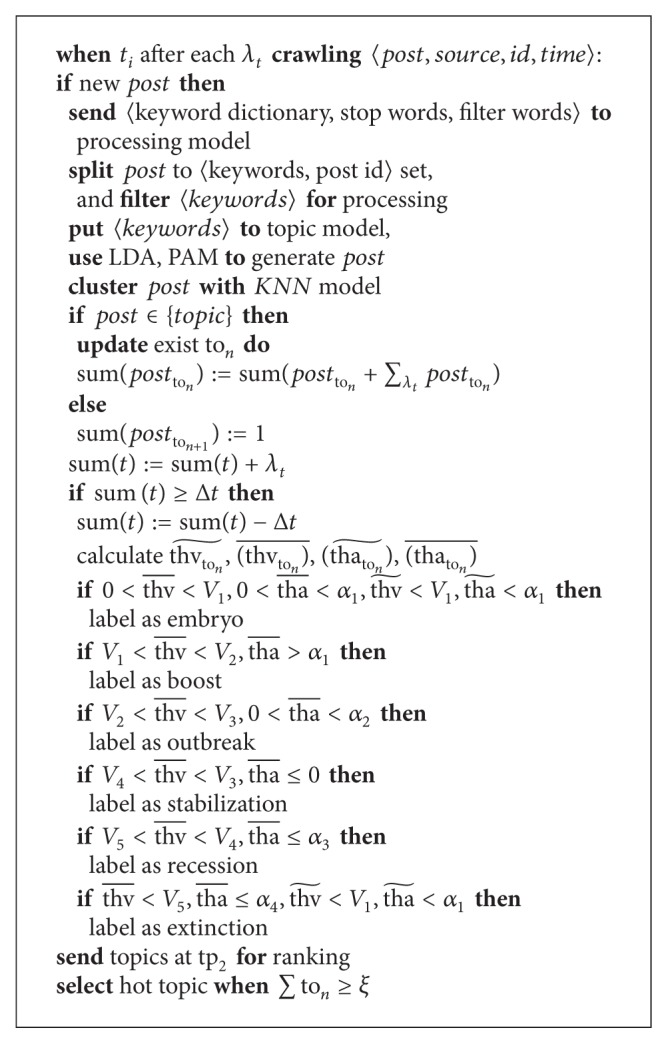


**Table 1:** The words distributed over five sample topics.

Latent topic	Words
Ukraine crisis	Independence, military, separate, control, flight, formation, revolution, illegal, occupy, territory
Search MH370	Search, plane, hope, missing, signal, hunt, batteries, clues, ocean, expiry
MH370 missing	Floating, objects, disappearance, hijack, radar, black hole, handover, duty, monitor, emergency
Spring Festival	National day, parade, festival, travel, vacation, food, government, ticket, relax, shopping
Crimea independence	Federation, referendum, vote, independence, celebration, join, republic, rename, division, council

**Table 2:** Hot topic ranks with related metrics.

Topic name	Post number	Subtopic number	Topic level	Recall (%)	Precision (%)
Flight MH370	122,012	57	6	79.54	81.25
Ukraine crisis	108,927	62	7	81.32	80.45
Crimea independence	89,877	48	5	70.55	76.24
South Korea ferry	77,092	55	6	74.21	76.98
Airpocalypse	65,768	36	4	72.36	75.78
Two sessions	62,182	40	5	77.25	81.32
I am a singer	50,653	48	5	66.16	72.65
Spring Festival	43,867	50	4	80.18	75.26
Syria Civil War	39,372	39	4	72.75	80.58
Taste of China	38,892	43	5	78.02	84.80

**Table 3:** Topic recognition time with topic hotness.

	European debt crisis	Syria	Food safety
Predict time	3Δ*t*	2Δ*t*	2Δ*t*
Google	10Δ*t*	8Δ*t*	12Δ*t*
Baidu	8Δ*t*	8Δ*t*	7Δ*t*
Topic amount	12,560	24,213	18,831
thv	6,500	9,327	18,831
tha	1,210	1,132	13,295
